# Impact of Obesity on Propofol Dosing: Implications for Anesthetic Outcomes

**DOI:** 10.7759/cureus.110550

**Published:** 2026-06-09

**Authors:** John Sawires, Shafayath Chowdhury, Cassie R Girardin, George Vega, Brandon Weissman, Varun Soti

**Affiliations:** 1 Anesthesiology and Critical Care, Lake Erie College of Osteopathic Medicine, Elmira, USA; 2 Neurology, Lake Erie College of Osteopathic Medicine, Elmira, USA; 3 Physical Medicine and Rehabilitation, Lake Erie College of Osteopathic Medicine, Elmira, USA; 4 Otolaryngology, Lake Erie College of Osteopathic Medicine, Elmira, USA; 5 Pharmacology and Therapeutics, Lake Erie College of Osteopathic Medicine, Elmira, USA

**Keywords:** anesthesia outcomes, intravenous anesthesia, lean body weight, obesity, pharmacodynamics (pd), pharmacokinetics (pk), propofol, propofol-induced anesthesia

## Abstract

Obesity presents significant challenges in anesthetic management because physiologic and body composition changes can substantially alter drug pharmacokinetics (PK) and pharmacodynamics (PD). Propofol is one of the most commonly used intravenous anesthetic agents for procedural sedation and general anesthesia; however, optimal dosing strategies in patients with obesity remain controversial. Conventional dosing based on total body weight (TBW) may increase the risk of adverse events, whereas alternative weight scalars and model-informed approaches have been proposed. This systematic review aimed to evaluate the impact of obesity on propofol dosing and propofol-associated anesthetic outcomes, with particular emphasis on hypnotic adequacy, hemodynamic stability, respiratory safety, recovery characteristics, and PK/PD considerations. This systematic review was conducted in accordance with the Preferred Reporting Items for Systematic Reviews and Meta-Analyses (PRISMA) 2020 guidelines. A comprehensive literature search was performed using PubMed, BioMed Central, and ClinicalTrials.gov. Eligible studies included randomized controlled trials, prospective and retrospective observational studies, and PK/PD investigations evaluating propofol administration in adults with obesity. Primary outcomes were hypnotic adequacy and hemodynamic stability. Secondary outcomes included respiratory and airway events, recovery characteristics, and PK/PD parameters. Risk of bias was assessed using the Joanna Briggs Institute Critical Appraisal Tools. Owing to substantial heterogeneity in study design, patient populations, dosing strategies, and outcome reporting, findings were synthesized using a structured narrative approach. Sixteen studies met the eligibility criteria and were included in the qualitative synthesis. The included studies demonstrated that obesity significantly influences propofol pharmacology and clinical response. Across studies, TBW-based dosing was frequently associated with greater hemodynamic depression, including hypotension and bradycardia, without consistently improving hypnotic adequacy. Respiratory and airway complications, particularly hypoxemia and the need for airway interventions, occurred more frequently in patients with higher body mass indexes during procedural sedation. PK/PD investigations consistently reported alterations in volume of distribution, clearance, and concentration-effect relationships in obese patients, supporting the use of lean body weight-based or model-informed dosing approaches. Recovery outcomes were less frequently reported, but when reported, generally indicated improved recovery profiles with reduced propofol requirements through individualized dosing strategies or adjunctive agents. Current evidence indicates that obesity substantially alters propofol’s PK, PD, and clinical anesthetic outcomes. TBW-based dosing may increase the risk of hemodynamic and respiratory adverse events without reliably improving anesthetic effectiveness. The available evidence supports considering alternative weight-scaling factors, individualized dose titration, and physiologic monitoring to optimize propofol administration in adults with obesity. Further prospective studies are needed to establish standardized dosing strategies and validate clinically applicable model-informed approaches.

## Introduction and background

Propofol remains one of the most widely used intravenous hypnotic agents for induction and maintenance of general anesthesia and for procedural sedation, owing to its rapid onset, favorable recovery profile, and predictable concentration-effect relationships. Since its initial development and early clinical adoption, propofol has become a cornerstone of modern anesthetic practice, supported by extensive pharmacologic characterization and decades of clinical experience. Regulatory approval and subsequent clinical pharmacology work have clarified its context-sensitive behavior, dose-response properties, and the central role of effect-site concentration in determining hypnotic depth. Nevertheless, even in general populations, propofol demonstrates substantial interindividual variability in both pharmacokinetics (PK) and pharmacodynamics (PD), influenced by age, comorbidity, and physiologic state. These sources of variability become particularly consequential in patient groups in whom drug distribution and clearance differ from the assumptions embedded in conventional dosing strategies, raising the risk that standard approaches may lead to either inadequate hypnosis or excessive cardiorespiratory depression [1-12].

Obesity represents one such clinical context in which propofol dosing becomes especially challenging. Although obesity is commonly defined and classified using body mass index (BMI), it is increasingly recognized that BMI alone does not fully capture the heterogeneity of body composition, fat distribution, and lean mass that influence drug behavior. Contemporary perspectives emphasize both the utility and the limitations of BMI as a clinical and research metric, particularly when weight-based dosing is used to guide administration of potent anesthetic agents. For anesthesiologists, the practical concern is not obesity as an epidemiologic construct but rather how excess adiposity and altered body composition modify the relationship between administered dose, plasma and effect-site concentrations, and clinical effect. A brief, targeted understanding of these classification issues is therefore necessary to contextualize dosing strategies without allowing background considerations to overshadow the primary pharmacologic question [13-18].

From a mechanistic standpoint, obesity can alter propofol disposition through multiple pathways. Increases in fat mass expand the apparent volume of distribution for lipophilic agents. In contrast, changes in lean body mass, cardiac output, and regional blood flow may influence both distribution kinetics and clearance. These physiologic shifts challenge the validity of simple total body weight (TBW)-based dosing, particularly for induction boluses, where excessive initial exposure may precipitate hypotension, bradycardia, or prolonged apnea. Pharmacometric investigations have demonstrated that obesity is associated with measurable differences in propofol PK and have proposed population models that better describe drug behavior in morbidly obese patients. In parallel, practical dosing guidance has increasingly favored alternative scalars, such as lean body weight (LBW) or model-based allometric approaches, to reduce the risk of overshoot while preserving adequate hypnotic depth [11,19-24].

These pharmacologic considerations are not merely theoretical. Clinical anesthetic care in patients with obesity is frequently complicated by reduced cardiopulmonary reserve and increased sensitivity to the dose-dependent depressant effects of hypnotics. Respiratory physiology is commonly altered, with reduced functional residual capacity, greater propensity for airway closure, and higher prevalence of obstructive sleep apnea and related hypoventilation syndromes. Contemporary reviews and practice-oriented guidance highlight that these features increase the likelihood of peri-induction hypoxemia, difficult ventilation, and postoperative respiratory compromise, particularly when sedative and hypnotic agents are not carefully titrated. In this setting, both underdosing and overdosing carry meaningful risks: inadequate hypnosis may lead to patient movement or awareness, whereas excessive dosing may exacerbate hemodynamic instability and prolong recovery [25-32].

Cardiovascular effects are similarly relevant. Propofol is well known to produce dose-dependent reductions in systemic vascular resistance and myocardial contractility, effects that can be magnified in patients with limited hemodynamic reserve. Obesity-related cardiac adaptations and comorbid disease may further narrow the therapeutic window, making precise dosing and vigilant monitoring essential. Perioperative management frameworks for obese patients consistently emphasize individualized anesthetic planning, careful titration of intravenous agents, and anticipation of exaggerated physiologic responses to standard doses. Within this broader clinical context, the choice of weight scaling and dosing strategy for propofol is a critical determinant of intraoperative stability and early postoperative recovery [26,33-36].

Objective monitoring of hypnotic depth has been proposed as one strategy to mitigate dosing uncertainty in high-risk populations. Processed electroencephalographic indices, such as the bispectral index (BIS), have been widely studied as tools to support titration of anesthetic agents and to reduce the likelihood of both awareness and excessive dosing. Although no monitoring modality substitutes for clinical judgment, depth-of-anesthesia monitoring provides a physiologic anchor that may be particularly valuable when conventional dose-effect relationships are altered, as in obesity. Prior work has explored correlations between clinical sedation scales and BIS values. It has reviewed the strengths and limitations of available monitoring devices, reinforcing the concept that integrating pharmacologic dosing with real-time physiologic feedback may improve safety margins in vulnerable patient groups [37-40].

The clinical literature addressing propofol use in patients with obesity spans both procedural sedation and operative anesthesia. In the endoscopic and procedural setting, several observational studies and cohort analyses have examined sedation-related complications associated with propofol and have specifically evaluated obesity as a risk factor for adverse events. These investigations have reported variable rates of hypoxemia, airway intervention, and hemodynamic instability, suggesting that patient habitus and dosing strategy may meaningfully influence outcomes. More recent work has explored target-controlled infusion approaches and adjunctive regimens, including randomized comparisons that incorporate agents such as esketamine to improve procedural conditions while limiting propofol exposure. Collectively, these studies underscore that obesity modifies the risk profile of propofol-based sedation, but they also highlight substantial heterogeneity in study design, outcome definitions, and monitoring practices [41-49].

In the operating room, additional studies have focused on induction dosing, PK modeling, and hemodynamic effects of propofol in obese and morbidly obese patients. Early investigations questioned whether TBW is an appropriate predictor of maintenance dose, while others examined the influence of body compartments on induction requirements. Subsequent PK modeling work provided more formal evidence that obesity alters both distribution and clearance, leading to recommendations for alternative dosing strategies. More recent clinical comparisons have evaluated TBW- versus LBW-based dosing in bariatric surgery and other settings, reporting differences in hemodynamic stability and depth of anesthesia. Despite these advances, the evidence base remains fragmented, with variable endpoints, inconsistent monitoring practices, and limited integration of pharmacometric and clinical outcome data [20,23,45,46,50-52].

From an evidence synthesis perspective, these limitations complicate translating individual study findings into coherent clinical guidance. Systematic reviews in adjacent areas of anesthetic practice have emphasized the importance of transparent reporting, well-defined outcomes, and rigorous appraisal of study quality. Contemporary reporting standards, such as Preferred Reporting Items for Systematic Reviews and Meta-Analyses (PRISMA) 2020, provide a framework for reproducible evidence synthesis. In contrast, established hierarchies of evidence and critical appraisal tools support nuanced interpretation of heterogeneous data. Applying these principles to the literature on propofol dosing in obesity is essential for distinguishing consistent signals from context-specific findings and identifying areas where current evidence is insufficient to support firm recommendations [53-55].

Against this background, a targeted synthesis focusing on propofol-specific anesthetic outcomes in patients with obesity is warranted. Importantly, such a synthesis should avoid recapitulating the broader epidemiology or long-term complications of obesity and instead concentrate on the peri-anesthetic questions that drive daily clinical decision-making: how dosing strategy influences hypnotic adequacy, hemodynamic stability, respiratory safety, and recovery profiles. For this review, “anesthetic outcomes” are defined as clinical endpoints that reflect the effectiveness and safety of propofol administration, including measures of hypnotic depth during induction and maintenance, occurrences of hypotension or bradycardia, respiratory or airway events, and early recovery characteristics, such as time to emergence or extubation, when reported. Framing outcomes in this manner aligns the review with clinically meaningful decisions faced by anesthesiologists and proceduralists [11,21,23,56,57].

The objective of this systematic review is to synthesize the available clinical evidence on the impact of obesity on propofol dosing and propofol-associated anesthetic outcomes in adult patients. Primary outcomes were measures of hypnotic adequacy and hemodynamic stability during anesthesia induction and maintenance. In contrast, secondary outcomes included respiratory or airway events, total propofol dose requirements, recovery characteristics such as time to emergence or extubation, and reported PK or PD parameters. To address the substantial heterogeneity across study designs, populations, dosing strategies, and outcome definitions, findings were synthesized thematically across clinically relevant domains rather than pooled quantitatively. By consolidating these data within a structured and transparent framework, this review aims to clarify current evidence, highlight consistent patterns and important uncertainties, and inform future research directions toward more individualized, physiology-based dosing strategies for propofol in patients with obesity.

## Review

Methods

Protocol and Reporting Standards

The review protocol was registered with the International Prospective Register of Systematic Reviews (PROSPERO) under registration number CRD420251160881. This systematic review was conducted and reported in accordance with the PRISMA guidelines to ensure transparent identification, selection, appraisal, and synthesis of studies [53].

Eligibility Criteria

Clinical studies evaluating the use of propofol in adult patients with obesity undergoing procedural sedation or general anesthesia were included. Studies were considered eligible if at least one clinically relevant anesthetic outcome related to propofol administration was reported, including measures of hypnotic depth, hemodynamic stability, respiratory or airway events, or early recovery characteristics. Interventional and observational study designs were considered, including randomized controlled trials, prospective and retrospective cohort studies, and comparative observational studies, consistent with standard evidence frameworks used in clinical research synthesis [53,54].

Case reports, narrative reviews, editorials, commentaries, and studies that did not report original clinical data were excluded. Studies focused exclusively on pediatric populations, or that did not report outcomes specifically related to propofol, were also excluded. When multiple publications reported overlapping populations, the most comprehensive or most recent report was retained [53,54].

Databases and Search Strategy

A comprehensive literature search was performed in PubMed, BioMed Central, and ClinicalTrials.gov to identify relevant studies evaluating propofol use in patients with obesity. The search strategy combined Medical Subject Headings (MeSH) and free-text terms related to obesity, propofol, anesthesia, sedation, and clinical outcomes. Searches were conducted from database inception through the most recent update prior to manuscript submission, and reference lists of included studies were screened to identify additional eligible articles [53,54].

The full, reproducible search strings for each database are provided in the Appendix. In brief, the PubMed strategy included combinations of MeSH terms such as “obesity”, “BMI”, “propofol”, “sedation”, “anesthesia”, and terms capturing intraoperative and postoperative complications, with database-specific syntax adapted as needed [53,54].

Study Selection

All records retrieved from the searches were imported into a reference management system, and duplicate entries were removed. Two reviewers independently screened titles and abstracts for potential eligibility, followed by duplicate full-text assessment according to predefined criteria. Discrepancies at any stage were resolved through discussion and, when necessary, consultation with a third reviewer [53,54].

Data Extraction

Data were extracted independently by two reviewers using a standardized data collection form. Extracted variables included study design, sample size, patient characteristics, definitions of obesity, clinical setting (procedural sedation or general anesthesia), propofol dosing strategy and weight scalar used (such as TBW, LBW, or model-based dosing), use of adjunct agents, monitoring strategies (including processed electroencephalographic indices when reported), and reported outcomes [11,21-23,37,40].

Outcome data extracted included measures of hypnotic depth, hemodynamic variables (such as hypotension or bradycardia), respiratory or airway events, total propofol dose requirements, and recovery characteristics such as time to emergence or extubation. Key PK or PD findings were also recorded when available. Disagreements in data extraction were resolved by consensus [11,20-22,41,42].

Outcomes

The primary outcomes of interest were measures of anesthetic effectiveness and safety following propofol administration in patients with obesity. These included indicators of hypnotic depth during induction or maintenance, as well as hemodynamic instability, such as hypotension or bradycardia [11,37,40,46].

Secondary outcomes included respiratory or airway events, total propofol dose requirements, and early recovery characteristics, including time to emergence or extubation. When reported, PK/PD parameters related to propofol disposition and effect were also extracted and summarized [11,20-22,25,58].

Risk of Bias Assessment

Methodological quality and risk of bias of included studies were assessed using the revised Joanna Briggs Institute Critical Appraisal Tools appropriate to each study design. Two reviewers independently performed the assessments, and disagreements were resolved through discussion. A study-level summary of risk-of-bias judgments was prepared to support transparent interpretation of the evidence [55].

Data Synthesis

Given the heterogeneity in study designs, patient populations, dosing strategies, and outcome definitions, a quantitative meta-analysis was not feasible. Instead, a structured narrative synthesis was conducted, aligned with PRISMA reporting standards for systematic reviews when statistical pooling is not suitable [53].

Studies were grouped into thematic domains based on their primary focus, including (1) induction dosing and hypnotic depth, (2) hemodynamic effects, (3) respiratory or airway events, (4) recovery characteristics, and (5) PK or PD modeling. Within each domain, findings were compared qualitatively across studies. When available, basic quantitative descriptors (for example, ranges, means, and proportions) were summarized to provide context. Consistency and divergence of findings were examined, and plausible sources of heterogeneity were explored descriptively [11,20-22,41,42,45,46,52].

Results

Study Selection

**Figure 1:**
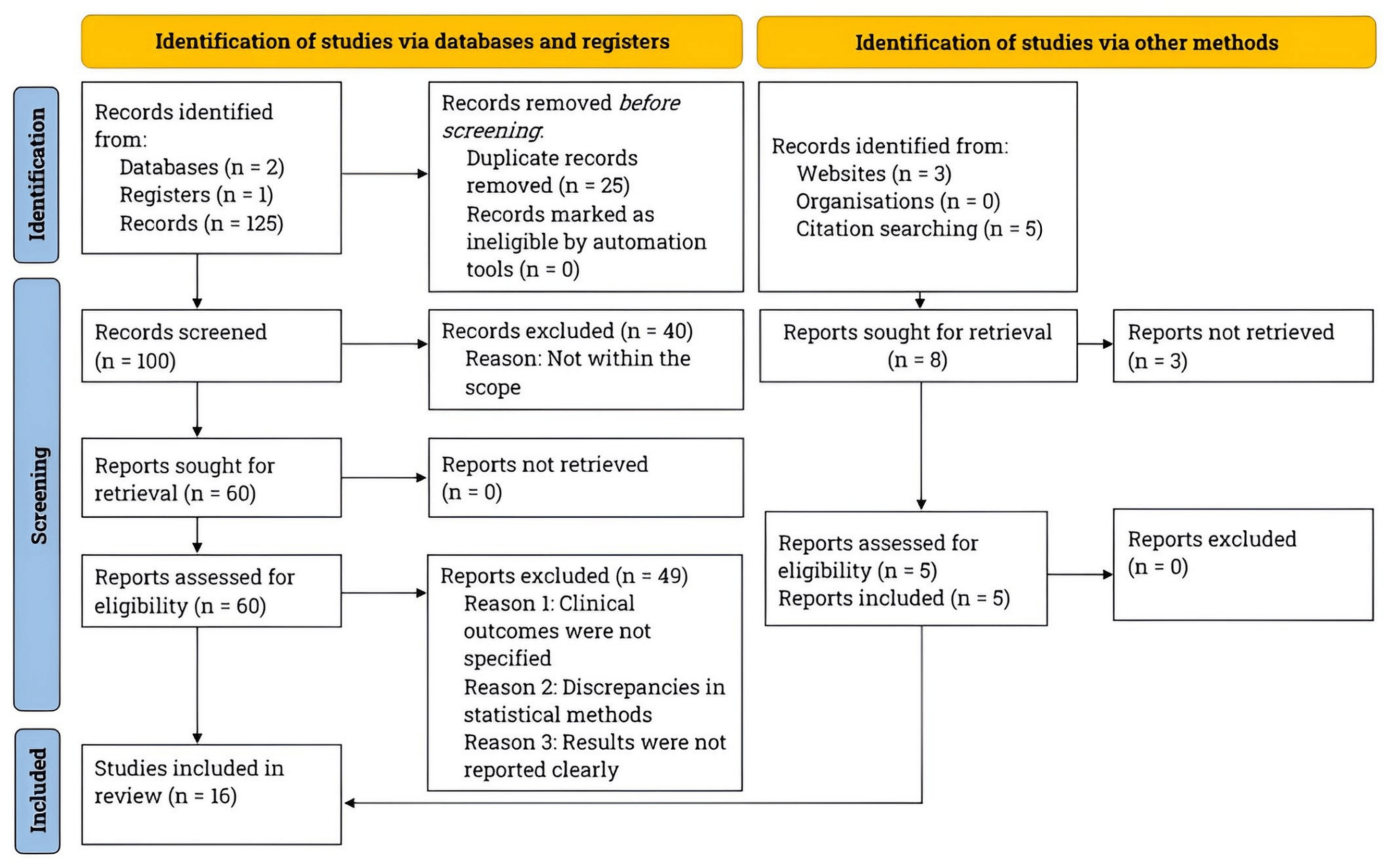
PRISMA flowchart depicting the literature search and study selection process PRISMA guidelines were followed. A thorough search was conducted using the databases PubMed and BioMed Central and the ClinicalTrials.gov registry, focusing on studies related to propofol and obesity: obesity and its epidemiology, surgical considerations in obese patients, propofol dosing in obese patients, and the impact of obesity on the outcomes of propofol-induced anesthesia. The total number of included studies is 16 (nine clinical outcome studies and seven PK/PD/modeling studies). n: number, PD: pharmacodynamics, PK: pharmacokinetics, PRISMA: Preferred Reporting Items for Systematic Reviews and Meta-Analyses

The search and screening process identified a body of literature examining the relationship between obesity and propofol-associated anesthetic outcomes across procedural sedation and operative settings. A total of 16 studies met the inclusion criteria, consisting of nine clinical outcome studies and seven PK/PD or modeling studies. The final included studies comprised randomized controlled trials, prospective and retrospective cohort studies, and PK/PD modeling investigations. These studies collectively evaluated hypnotic depth, hemodynamic stability, respiratory and airway events, recovery characteristics, and PK or PD parameters. The study selection process is summarized in the PRISMA flow diagram (Figure 1).

Study Characteristics

A substantial proportion of the clinical studies were conducted in the context of advanced endoscopic procedures, with others performed in surgical populations, including patients undergoing bariatric procedures. Obesity definitions and patient stratification strategies varied, most commonly relying on BMI thresholds. Propofol dosing strategies differed across studies and included TBW dosing, LBW-based dosing, and model-informed approaches. Several studies incorporated processed electroencephalographic monitoring, while others relied on clinical endpoints to assess hypnotic adequacy. Reported outcomes included sedation-related complications, hemodynamic changes, respiratory events, total propofol dose requirements, and recovery parameters, underscoring the heterogeneity of both study design and outcome reporting. Key characteristics of the included studies are summarized in Table 1.

**Table 1:** Key characteristics of included studies BP: blood pressure, EC50: half maximal effective concentration, ED95: effective dose in 95% of patients, LBW: lean body weight, OR: operating room, PD: pharmacodynamics, PK: pharmacokinetics, RCT: randomized controlled trial, SRCs: sedation-related complications, TBW: total body weight, TCI: target-controlled infusion, vs: versus [41-49]

Study authors	Design	Setting	Population	Dosing strategy	Key outcomes
Coté et al. (2010) [41]	Controlled clinical trial	Endoscopy	Adults	Propofol sedation	Airway modifications, hypoxemia, hypotension
Wani et al. (2011) [42]	Prospective cohort	Endoscopy	Adults	Propofol sedation	SRCs, airway maneuvers, hypoxemia
Olutoye et al. (2012) [43]	Controlled study	OR	Children	Induction dosing	ED95, BP changes
Chidambaran et al. (2013) [44]	Observational	OR	Children and adolescents	Propofol anesthesia	Respiratory events, dose requirements
Dong et al. (2016) [45]	PK/PD study	OR	Adults	TBW vs LBW	PK parameters, EC50
Wu et al. (2018) [46]	Clinical study	OR	Adults	TBW vs LBW	Hemodynamic effects
Kilic et al. (2019) [47]	Retrospective	Endoscopy	Adults	Ketofol	Airway events, complications
García Guzzo et al. (2020) [48]	Retrospective cohort	Endoscopy	Adults	TCI propofol	Desaturation, hypotension, bradycardia
Zheng et al. (2023) [49]	RCT	Endoscopy	Adults	Propofol with and without esketamine	Dose, hemodynamics, adverse events

Risk of Bias

Overall, randomized, prospective studies generally demonstrated a low risk of selection bias, although blinding and allocation concealment were variably reported. Observational and retrospective studies were more prone to detection and reporting bias, largely due to subjective outcome assessment and incomplete control for confounding variables. Despite these limitations, most studies were judged to be of sufficient methodological quality for qualitative synthesis, though the heterogeneity of designs, dosing protocols, and outcome measures precluded quantitative meta-analysis. Risk-of-bias assessment using the Joanna Briggs Institute Critical Appraisal Tools is summarized in Table 2.

**Table 2:** Risk of bias summary The risk of bias of the included studies was assessed using the revised Joanna Briggs Institute Critical Appraisal Tools. [41-49]

Study authors	Selection bias	Performance bias	Detection bias	Reporting bias	Overall
Coté et al. (2010) [41]	Low	Moderate	Moderate	Low	Moderate
Wani et al. (2011) [42]	Low	Moderate	Moderate	Low	Moderate
Olutoye et al. (2012) [43]	Low	Moderate	Moderate	Low	Moderate
Chidambaran et al. (2013) [44]	Moderate	Moderate	Moderate	Moderate	Moderate
Dong et al. (2016) [45]	Low	Low	Low	Low	Low
Wu et al. (2018) [46]	Low	Low	Low	Low	Low
Kilic et al. (2019) [47]	Moderate	Moderate	Moderate	Moderate	Moderate
García Guzzo et al. (2020) [48]	Moderate	Moderate	Moderate	Moderate	Moderate
Zheng et al. (2023) [49]	Low	Low	Low	Low	Low

Synthesis of Findings by Thematic Domain

Induction dosing and hypnotic adequacy: Evidence from both adult and pediatric populations shows that obesity affects propofol dose-response relationships. In pediatric patients, obesity was associated with a lower effective dose (ED95) for loss of consciousness and greater decreases in blood pressure after induction, suggesting increased central nervous system sensitivity to propofol [43]. PK studies also demonstrated that TBW-based dosing led to altered plasma concentration profiles, increased clearance and volume of distribution, and lower half maximal effective concentration (EC50) values, supporting the use of LBW or model-based dosing to prevent excessive anesthesia depth [45,46]. Overall, these findings suggest that standard weight-based dosing strategies may not reliably predict hypnotic effects in patients with obesity.

Hemodynamic stability: Several studies consistently report a higher incidence of hemodynamic instability in patients with obesity receiving propofol, especially when dosing is based on TBW. Cardiovascular depression, including hypotension and bradycardia, is more significant with higher propofol doses, while LBW-based dosing strategies show a comparatively safer PD profile [46]. In a randomized trial assessing the addition of esketamine to propofol for gastroscopy in patients with obesity, the adjunct therapy reduced propofol requirements and enhanced hemodynamic stability, further emphasizing propofol's dose-dependent cardiovascular effects in this population [49].

Respiratory/airway events: Respiratory and airway complications are among the most consistently reported adverse outcomes linked to propofol use in patients with obesity. In large cohorts undergoing advanced endoscopic procedures, a higher BMI was independently associated with increased rates of airway adjustments and hypoxemia [41,42]. The incidence of airway maneuvers and oxygen desaturation increased progressively across higher BMI categories, and obesity combined with higher BMI class, as defined by the American Society of Anesthesiologists, further elevated risk [42]. Additional studies reported higher rates of apnea, desaturation, and airway obstruction in patients with higher BMIs, highlighting the clinical vulnerability of this population during propofol sedation [47,48].

Recovery characteristics: Recovery-related outcomes are reported less consistently but suggest a link between cumulative propofol exposure and delayed emergence. In studies comparing different sedation strategies, lower propofol requirements, such as when combined with esketamine, were associated with shorter induction and awakening times and higher provider satisfaction [49]. These findings indicate that dose-sparing approaches may improve recovery profiles in patients with obesity, although broader validation across surgical settings remains limited.

PK/PD modeling: Mechanistic studies revealed that obesity significantly affects propofol’s PK/PD, resulting in increased volume of distribution and clearance and modifying concentration-effect relationships. Population modeling in morbidly obese patients undergoing bariatric surgery confirmed that TBW is an unreliable predictor of propofol exposure and effect, supporting the use of alternative weight measures or model-based dosing strategies to minimize the risk of overdosing during anesthesia induction and maintenance [22,45,46]. These findings provide a physiological foundation for the clinical observations seen across procedural sedation and surgical settings. Please refer to Table 3, summarizing these key outcomes by thematic domain.

**Table 3:** Key outcomes by thematic domain ↑: increased, BMI: body mass index, BP: blood pressure, CV: cardiovascular, EC50: half-maximal effective concentration, D95: effective dose in 95 percent of patients, PD: pharmacodynamics, PK: pharmacokinetics, SRCs: sedation-related complications, Vd: volume of distribution. “-” indicates outcome not reported or not assessed in the study.

Study authors	Hypnotic depth	Hemodynamics	Respiratory/airway	Recovery	PK/PD
Coté et al. (2010) [41]	-	Hypotension rare	Increased airway modalities, hypoxemia	-	-
Wani et al. (2011) [42]	-	-	Increased SRCs with BMI	-	-
Olutoye et al. (2012) [43]	Decreased ED95	Decreased BP post-induction	-	-	-
Chidambaran et al. (2013) [44]	-	-	Increased respiratory adverse events	Prolonged sedation	-
Dong et al. (2016) [45]	Altered EC50	-	-	-	↑ Vd, ↑ clearance
Wu et al. (2018) [46]	-	Increased CV depression	-	-	Altered PD
Kilic et al. (2019) [47]	Increased Induction time	-	Increased airway interventions	-	-
García Guzzo et al. (2020) [48]	-	Hypotension, bradycardia	Increased desaturation	-	-
Zheng et al. (2023) [49]	-	Improved with esketamine	Reduced adverse events	Faster recovery	-

Discussion

This systematic review synthesizes clinical and pharmacologic evidence on propofol dosing and anesthetic outcomes in adults with obesity, focusing on hypnotic adequacy, hemodynamic stability, respiratory safety, recovery characteristics, and PK/PD considerations. When the evidence is organized thematically rather than as isolated study summaries, consistent patterns emerge despite significant heterogeneity in study design, settings, dosing strategies, and outcome definitions. Across procedural sedation and operative anesthesia, obesity influences the relationship among administered dose, achieved effect, and physiologic tolerance, and TBW-based dosing has been repeatedly linked to higher rates of adverse events without clear improvement in anesthetic adequacy [11,20-22,41-49,52].

Findings related to induction dosing and hypnotic depth show that obesity changes propofol dose-response relationships in a way that challenges the established practice of dosing based on weight-based scaling. Mechanistic and clinical studies consistently reveal shifts in concentration-effect relationships, indicating lower effective-dose thresholds and altered distribution kinetics in obese groups. These findings explain why TBW-based dosing can lead to excessive early exposure during induction and support the use of LBW or model-informed approaches as better starting points for dosing in this population [11,20-23,45,46,50,51].

Hemodynamic outcomes are among the most consistent clinical signals in the reviewed literature. Higher propofol doses, especially when calculated using TBW, are linked to increased rates of hypotension and bradycardia during induction and early maintenance. Considering the known cardiovascular effects of propofol, these findings are clinically significant in patients with obesity, who may have diminished cardiovascular reserve. Comparative studies and trials show that dose-sparing strategies, including alternative weight scalars or adjunctive agents, can lower propofol requirements and enhance hemodynamic stability, arguing against routinely increasing induction doses based solely on TBW [11,21,45,46,49,52].

Respiratory and airway events highlight the narrow therapeutic window in this population. Large cohorts undergoing advanced endoscopic procedures consistently show higher rates of hypoxemia, airway obstruction, and need for airway interventions as BMI increases. While underlying respiratory physiology contributes to this vulnerability, the reviewed studies also implicate sedation depth and cumulative propofol exposure as modifiable factors. These findings stress the importance of careful titration, vigilant monitoring, and, when appropriate, dose-sparing or multimodal strategies to reduce respiratory risks during propofol-based sedation and anesthesia [25,41-49].

Recovery characteristics were reported less consistently but offer additional clinical context. When evaluated, higher overall propofol exposure was associated with longer times to emergence or readiness for discharge. In contrast, strategies that reduced propofol requirements, such as adjunctive agents, were associated with shorter induction and recovery times. Although recovery endpoints are affected by various perioperative factors, these data support the broader trend seen across domains: that excessive dosing has downstream costs beyond immediate cardiorespiratory effects [11,49,56,57].

The PK/PD modeling literature demonstrates a mechanistic link between these clinical observations. Population models in patients with obesity consistently show that TBW is a poor predictor of propofol exposure and effect, while alternative scalars and model-based methods better account for altered body composition and distribution kinetics. Despite this, the application of these models in routine clinical practice varies, and prospective validation of specific dosing algorithms based on outcomes is limited. The growing availability of target-controlled infusion systems and depth-of-anesthesia monitoring provides a practical way to incorporate model-informed dosing with real-time physiological feedback [11,20-22,37,40,49].

Several methodological considerations weaken the strength of inference. The included studies vary greatly in design, populations, dosing protocols, and outcome definitions, and risk-of-bias assessment showed inconsistent reporting quality, especially among observational studies. These limitations prevented a quantitative meta-analysis and required a structured narrative synthesis. Nonetheless, agreement across mechanistic, interventional, and observational studies supports the conclusion that conventional TBW-based dosing is suboptimal in adults with obesity [41-49,53,55].

From a clinical perspective, the implications are practical rather than strict guidelines. The evidence does not support a single universal dosing formula for propofol in patients with obesity. Instead, it advocates for a framework that combines more suitable initial dosing, careful titration to effect, and physiologic monitoring to guide ongoing administration. This approach recognizes both altered propofol pharmacology in obesity and persistent interindividual variability, aligning with broader principles of individualized anesthesia care [11,21-23,37,40,52].

Future research should focus on prospective, sufficiently powered studies that compare dosing strategies using clinically significant endpoints, such as hemodynamic stability, respiratory safety, and recovery profiles. Enhancing the standardization of outcome definitions and reporting would improve comparability, and integrating PK/PD modeling more closely with clinical monitoring could accelerate translation into practice [11,20-22,56].

This review shows that obesity significantly impacts propofol pharmacology and increases the risk of hemodynamic and respiratory adverse effects when TBW-based dosing strategies are used. The combination of clinical and mechanistic evidence supports adopting alternative weight scalars or model-informed dosing, along with careful titration and monitoring, to improve the safety and effectiveness of anesthesia in this growing patient group [11,20-22,45,46,49,52].

## Conclusions

This systematic review shows that obesity alters the pharmacologic behavior of propofol and increases the risk of hemodynamic and respiratory adverse effects when using conventional TBW-based dosing. Clinical and mechanistic studies in procedural sedation and operative anesthesia demonstrate that the relationship between dose, hypnotic effect, and physiologic tolerance is nonlinear and highly variable in individuals with obesity. The evidence indicates that dosing based solely on body size is insufficient and may expose patients to unnecessary risks without reliably enhancing anesthetic outcomes. These findings support the use of alternative weight-scaling methods or model-informed dosing, combined with careful titration and physiologic monitoring, as more effective approaches for administering propofol in adults with obesity. Due to variability in study designs and outcome reporting, we cannot recommend a single dosing algorithm; however, the clinical and PK/PD data clearly suggest that we should move beyond simple TBW-based dosing. Future research should prospectively compare different dosing strategies, adopt standardized outcome measures, and incorporate model-informed dosing with real-time monitoring. This work is essential for translating mechanistic insights into practical, individualized anesthesia care and enhancing the safety and efficacy of propofol in this growing patient population.

## Appendices

Our objective was to identify and review publications that specifically examine the impact of obesity on propofol-related pharmacokinetics, pharmacodynamics, dosing, and anesthetic outcomes in obese patient populations. We systematically searched PubMed, BioMed Central, and clinicaltrials.gov for studies completed or published before June 20, 2025. Below are the complete search queries used in PubMed, BioMed Central, and ClinicalTrials.gov.

PubMed

(((“Obesity”[MeSH] OR “Body Mass Index”[MeSH] OR Obesity[TIAB] OR BMI[TIAB])) AND ((“Propofol”[MeSH] OR Propofol[TIAB])) AND ((“Models, Theoretical”[MeSH] OR “Pharmacokinetics”[MeSH] OR “PK Modeling”[TIAB] OR “PD Modeling”[TIAB] OR “Lean Body Weight”[TIAB] OR LBM[TIAB] OR “Total Body Weight”[TIAB] OR TBW[TIAB] OR “Dosing”[TIAB]))) AND ((“Consciousness Monitors”[MeSH] OR “Hypnotic adequacy”[TIAB] OR “sedation outcomes”[TIAB] OR “Respiratory Events”[TIAB] OR “Airway Events”[TIAB] OR “Hemodynamic Instability”[TIAB] OR “Recovery characteristics”[TIAB]))

BioMed Central

(Obesity OR BMI OR “Body Mass Index”) AND (Propofol) AND (“Pharmacokinetic Modeling” OR “Pharmacodynamic Modeling” OR “Lean Body Weight” OR LBM OR “Total Body Weight” OR TBW OR Dosing) AND (“Hypnotic Adequacy” OR “Sedation Outcomes” OR “Respiratory Events” OR “Airway Events” OR “Hemodynamic Instability” OR “Recovery Characteristics”)

Clinicaltrials.gov

AREA[ConditionSearch](Obesity OR BMI OR “Body Mass Index”)

AND AREA[InterventionSearch](Propofol)

AND (“Pharmacokinetic Modeling” OR “Pharmacodynamic Modeling” OR “Lean Body Weight” OR LBM OR “Total Body Weight” OR TBW OR Dosing)

AND (“Hypnotic Adequacy” OR “Sedation Outcomes” OR “Respiratory Events” OR “Airway Events” OR “Hemodynamic Instability” OR “Recovery Characteristics”)

AND AREA[OverallStatus](Completed)
